# Editorial: Metabolism in Alzheimer's Disease

**DOI:** 10.3389/fnins.2021.824145

**Published:** 2022-01-04

**Authors:** Jill K. Morris, Levi B. Wood, Heather M. Wilkins

**Affiliations:** ^1^Department of Neurology, University of Kansas Medical Center, Kansas City, KS, United States; ^2^Department of Neurology, University of Kansas Alzheimer's Disease Center, Kansas City, KS, United States; ^3^Department of Molecular and Integrative Physiology and Internal Medicine-Division of Endocrinology, University of Kansas Medical Center, Kansas City, KS, United States; ^4^Parker H. Petit Institute for Bioengineering and Bioscience, Georgia Institute of Technology, Atlanta, GA, United States; ^5^The Wallace H. Coulter Department of Biomedical Engineering, Georgia Institute of Technology, Atlanta, GA, United States; ^6^George W. Woodruff School of Mechanical Engineering, Georgia Institute of Technology, Atlanta, GA, United States; ^7^Department of Biochemistry and Molecular Biology, University of Kansas Medical Center, Kansas City, KS, United States

**Keywords:** metabolism, mitochondria, Alzheimer's disease, mitophagy, inflammation

Alzheimer's disease (AD) pathology begins decades before clinical onset of dementia. Amyloid beta (Aβ) generally accumulates first in cognitively normal (CN) individuals, with tau and cognitive abnormalities following (Jack et al., 2013). AD pathologies have been found to correlate and interact with metabolic outcomes in studies spanning numerous experimental paradigms (Mosconi et al., 2009, 2010a,b,c; Mosconi, 2013; Morris et al., 2014a; Wilkins et al., 2014; Swerdlow et al., 2017; Weidling et al., 2020; Wilkins and Swerdlow, 2021).

Metabolic changes are prominent in AD. Fluorodeoxyglucose positron emission tomography (FDG-PET) comparing AD and CN individuals reveals lower glucose levels in the brains of AD patients (Herholz et al., 2002; Mosconi et al., 2010a; Marcus et al., 2014; Suppiah et al., 2019). These findings have led to overwhelming evidence of metabolic deficiencies in AD. Beyond reductions in brain glucose metabolism, mitochondrial dysfunction is observed not only within the brain but also systemically (Parker, 1991; Kish et al., 1992; Cardoso et al., 2004a,b; Morris et al., 2014b; Fisar et al., 2016; Guo et al., 2017; Swerdlow, 2018; Baloyannis, 2019; Chakravorty et al., 2019). More recent genome wide association studies (GWAS) identified risk-associated single nucleotide polymorphisms (SNPs) in genes which function in mitochondrial and metabolic pathways (Lakatos et al., 2010; Swerdlow et al., 2020; Harwood et al., 2021; Wightman et al., 2021). Apolipoprotein E (*APOE*), the strongest genetic risk factor for sporadic AD, is both central to lipid metabolism and has been found to interact with inherited mitochondrial genes to amplify risk for AD (Carrieri et al., 2001; Andrews et al., 2020; Swerdlow et al., 2020). Moreover, molecular studies of AD brain show an overall reduction in the number of intact mitochondria and mitochondrial DNA (Swerdlow, 2018; Wilkins and Swerdlow, 2021). Thus, mitochondrial function/dysfunction plays a role in protein aggregation, inflammation, and cell death; all events observed in AD. Overall, metabolism and mitochondrial function/dysfunction are strongly associated with AD.

The goal of this Research Topic was to further understand topics in the AD field that broadly focus on metabolic changes in AD and the interaction between metabolism, AD risk factors, and pathologies. These include: the role of genetic risk factors for sporadic AD (such as *APOE*) in non-cell autonomous functions, the intersection between metabolism and inflammation, the role of metabolism in protein aggregation, how current therapies target metabolism, inflammation, and protein aggregation, the role of novel metabolism/mitochondrial genes identified by GWAS in pathological mechanisms, the role of metabolism in the communication between neurons and glia in AD, how we can leverage existing model systems and develop better models to address questions of brain metabolism in the context of AD.

The articles of this Research Topic highlight a variety of reviews and original research which discuss and address topics of metabolism in AD (Figure 1). Several articles focus on potential AD therapeutics. These include a thorough review on the use of pioglitazone for AD treatment. Pioglitazone is a peroxisome proliferator-activated receptor gamma and alpha (PPAR-γ; PPAR-α) activator. PPARs are transcription factors critical for regulation of many pathways implicated in AD including insulin and glucose metabolism, lipid homeostasis, inflammation, tau and Aβ homeostasis, and mitochondrial function. The review by Saunders et al. discusses pre-clinical and clinical data with longitudinal observational studies revealing a positive impact of pioglitazone in AD and dementia onset in those at risk. The authors also discuss the dose-dependent effects and the caveats revealing future needs for further study into discrepancies found with placebo controlled blinded studies. Norowitz and Querfurth discusses mTOR regulation and drug targeting in AD. The authors focus on nuances for targeting mTOR in therapies including specificity for disease/region/and timing, pleiotropy, personalized therapy with relation to the effects of genetic factors, and the role of lifestyle factors and interventions.

**Figure 1:**
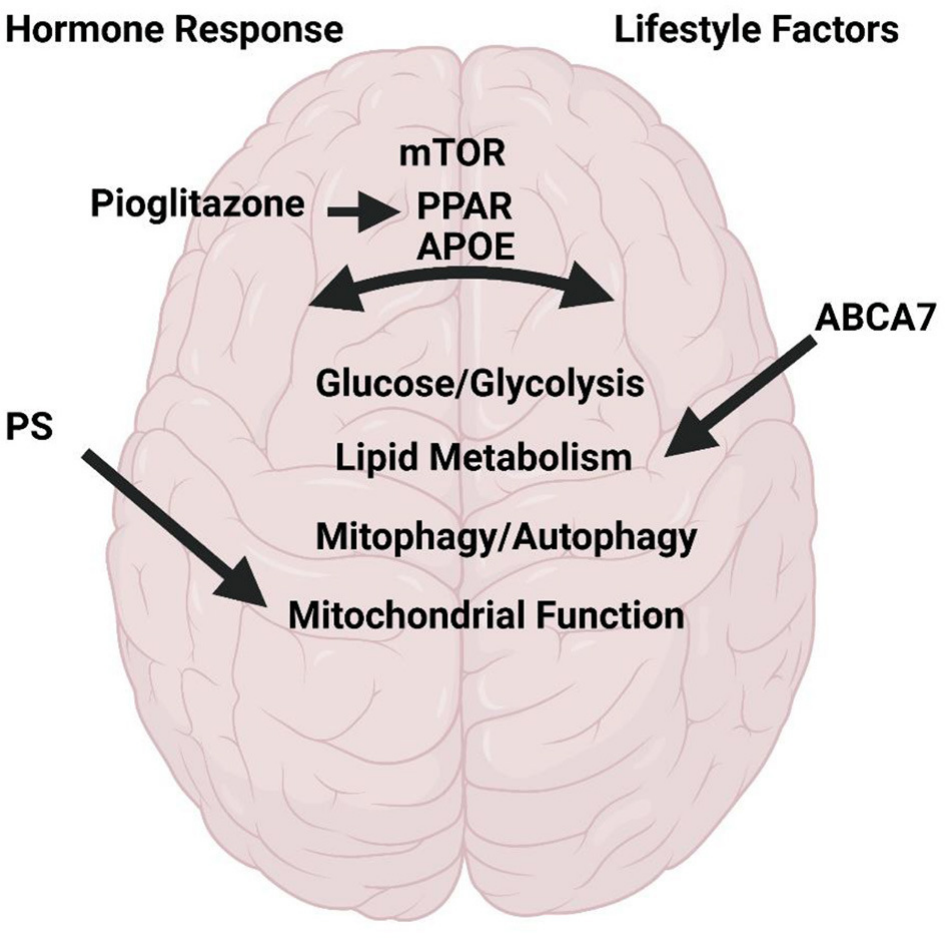
Metabolism in Alzheimer's Disease. Research and review articles discuss the role of hormone response to meals, lifestyle factors on brain function with respect to mTOR, PPAR, PS1, ABAC7, and APOE. All of which regulate glycolysis/glucose and lipid metabolism, mitophagy/autophagy, and mitochondrial function. Created with BioRender.com.

Several other articles discuss the role of specific metabolic pathways in AD. Zhang et al. reviewed the role of glycolytic metabolism in brain resilience in AD. The authors highlight the correlation between glycolytic flux, Aβ, and tau accumulation in humans, where decreased glycolytic function is associated with higher pathologies. In a separate review article, Kyrtata et al. discuss glucose transport in AD with particular focus on glucose transporter (GLUT) deficiencies in AD. The authors discuss the timing of changes to GLUT expression and glucose uptake in brain through rodent studies and how this relates to the timing of onset of Aβ pathology.

An additional review presents the effect of sialometabolism on brain health and AD. Rawal and Zhao discuss the role of sialic acids in brain function and neuroinflammation. The novelty of this pathway in AD is the identification of sialic acid binding Ig-like lectin 3 (CD33) as a genetic risk factor for AD through GWAS.

A separate AD genetic risk factor, ATP binding cassette subfamily A member 7 (ABCA7) was examined. Aikawa et al. used mice with haplodeficiency of ABCA7 to determine the response to immune modulation with lipopolysaccharide (LPS). The authors report that mice deficient in ABCA7 had activated lipid metabolism pathways. This study again highlights the relationship between metabolism and neuroinflammation. Morris et al. describes the role of meal stimulated hormone response through the incretin pathway in cognitive function and brain volume. The authors report that in human AD subjects, a higher meal-stimulated response of insulin, glucose, and peptide tyrosine was observed. Brain volume significantly correlated negatively with insulin, C-peptide, and glucose-dependent insulinotropic polypeptide (GIP). These articles highlight the role of diverse metabolic pathways in brain health, aging, and AD.

A focus on genetic risk factors and metabolism was discussed through a review of *APOE* in AD by Husain et al. The authors focused on the role of *APOE* in lipid transport and interactions with AD pathologies (such as tau and Aβ). Other genetic components of AD include mutations in presenilin (PS) in familial AD, and PS has a role in mitochondrial function. Contino et al. examined the role of PS deficiency on neurons and astrocytes derived from mice. Their prior studies showed mitochondrial deficits in mouse embryonic fibroblasts, but in the current study no effects were observed on similar endpoints. This study highlights the importance of model systems used for study.

Mitophagy and autophagy are implicated in AD and are the focus of many therapeutic initiatives. Tran and Reddy discuss deficiencies in autophagy and mitophagy in AD. The authors focus on metabolic drivers of autophagy/mitophagy deficiencies, the influence of aging, and how these pathways influence AD pathologies.

Collectively, the articles in this Research Topic emphasize that the field of brain metabolism in AD is emerging and generating large interest from a therapeutic standpoint. Progress in filling our gap in knowledge on the role of metabolism in AD will advance new therapeutic avenues for this devastating disease.

## Author Contributions

JM, LW, and HW contributed to the recruitment and editing of the special Research Topic. All authors contributed to writing and editing the editorial.

## Funding

Support for this project includes the Peg McLaughlin fund (JM and HW), R00AG050490 and R01AG062548 (JM), R01AG075820 (LW), R00AG056600 (HW), and P30AG072973.

## Conflict of Interest

The authors declare that the research was conducted in the absence of any commercial or financial relationships that could be construed as a potential conflict of interest.

## Publisher's Note

All claims expressed in this article are solely those of the authors and do not necessarily represent those of their affiliated organizations, or those of the publisher, the editors and the reviewers. Any product that may be evaluated in this article, or claim that may be made by its manufacturer, is not guaranteed or endorsed by the publisher.
